# Associations between personality disorder characteristics and treatment outcomes in people with co-occurring alcohol misuse and depression

**DOI:** 10.1186/s12888-016-0937-z

**Published:** 2016-07-07

**Authors:** Kristen L. McCarter, Sean A. Halpin, Amanda L. Baker, Frances J. Kay-Lambkin, Terry J. Lewin, Louise K. Thornton, David J. Kavanagh, Brian J. Kelly

**Affiliations:** School of Psychology, University of Newcastle, Callaghan, NSW 2308 Australia; School of Medicine and Public Health, University of Newcastle, Callaghan, NSW 2308 Australia; NHMRC Centre for Research Excellence in Mental Health and Substance Use, National Drug and Alcohol Research Centre, University of New South Wales, Sydney, NSW 2052 Australia; Centre for Brain and Mental Health Research, University of Newcastle, Callaghan, NSW 2308 Australia; Hunter New England Mental Health, PO Box 833, Newcastle, NSW 2300 Australia; Centre for Children’s Health Research, Institute of Health & Biomedical Innovation and School of Psychology & Counselling, Queensland University of Technology, Brisbane City, QLD 4000 Australia

**Keywords:** Personality disorder, Alcohol misuse, Depression, Comorbidity, Psychological interventions

## Abstract

**Background:**

Personality disorders are highly comorbid with alcohol misuse and depressive symptomatology; however, few studies have investigated treatment outcomes in this population. The aim of this study was to examine relationships between baseline personality disorder cluster profiles and overall and treatment-related changes for those with co-occurring alcohol misuse and depression.

**Methods:**

Secondary analysis was conducted using a subset of data (*N* = 290) from two randomised controlled trials of psychological interventions for co-occurring alcohol misuse and depression, which did not specifically target personality disorders. Baseline dimensional personality disorder cluster scores were derived from the International Personality Disorder Examination Questionnaire (IPDEQ). Four treatment conditions were compared: a brief integrated intervention, followed by no further treatment, or nine further sessions of integrated-, alcohol-, or depression-focused treatment. Associations between IPDEQ scores and changes in alcohol use, depressive symptoms and functioning from baseline to the 6- and the 12-month follow-ups were of primary interest.

**Results:**

Personality disorder cluster scores moderately negatively impacted on overall change (primarily Cluster C), as well as treatment-related outcomes (primarily Cluster A), particularly changes in depressive symptoms and psychosocial functioning. Longer interventions appeared to be more effective in the longer-term (e.g., at 12-month follow-up), with integrated interventions relatively more effective than single-focused ones for individuals with higher personality disorder cluster scores.

**Conclusions:**

Greater attention needs to be paid to particular personality disorder clusters during the assessment and treatment of individuals with co-occurring alcohol misuse and depression. Integrated interventions, incorporating motivational interviewing and cognitive behaviour therapy, may provide a useful therapeutic framework. Integrated interventions also provide opportunities for adjunctive components focussing on other issues and coping strategies (e.g., to offset negative affective states), potentially tailored to the characteristics and needs of individual participants.

## Background

### Comorbidity

Co-morbid alcohol misuse and mental health problems are a major health concern as they place significant burden on the health care system [1] and are associated with a broad range of negative outcomes, including more severe depressive symptoms, poorer social functioning, increased service utilisation, more days out of role, and poorer treatment outcomes [2, 3]. A weakness of previous comorbidity research is the tendency to focus on two co-occurring disorders only. For example, the associations between alcohol misuse and depressive symptoms have been widely studied [4]. However, research has not addressed more complex clinical presentations, such as where alcohol misuse, personality disorder and depressive symptoms are all present within the same individual [5]. Although prevalence estimates of personality disorders in the general population are approximately 6.5 % [6], estimates in mental health settings are much higher, ranging from 36 to 67 % [7]. Specifically, there appears to be an elevated rate of personality disorders among individuals with alcohol misuse [8, 9] and depression [10, 11].

Existing research on the co-occurrence of personality disorders and either alcohol misuse or depressive symptomatology consists mainly of prevalence based studies that do not address treatment outcomes [12, 13]. The existing studies are also limited by small sample sizes, inconsistent findings [14, 15], and failure to examine particular personality disorder cluster associations, even though these disorders are not expressed homogenously [16]. Gianoli et al. [17] reviewed pharmacological and psychotherapeutic treatment options for individuals with comorbid borderline personality disorder and alcohol misuse, concluding that there are currently few treatments that simultaneously address both sets of symptoms.

There is also ongoing debate about categorical versus dimensional approaches to the assessment of personality disorders [18], and about the relative merits of different assessment techniques [19]. We have previously expressed a preference for dimensional approaches [20], because they offer greater flexibility across clinical and non-clinical settings, and potentially facilitate a more integrative strategy for better characterising complex comorbidities [21].

### Interventions and treatment outcomes

Although it has been suggested by the World Health Organization that alcohol use disorders and major depression may require concurrent treatment [22], few randomized controlled trials e.g., [23–26] have used integrated psychological interventions for these co-occurring problems; see Kelly et al. [27] for a review of comorbid treatments for substance abuse and psychiatric conditions.

In the first study of its kind, Baker et al. [28] compared integrated (i.e., targeting both alcohol and depression) and single-focused (only alcohol or depression) outpatient treatment programs comprising of motivational interviewing (MI) and cognitive behaviour therapy (CBT) for alcohol misuse and/or depressive symptoms. Based on short-term, post-treatment outcomes (i.e., at 18-weeks), the authors reported that integrated treatment was associated with a reduction in drinking days and greater improvement in depressive symptoms. Although a dimensional screener for personality disorders was included in the baseline phase of this study, outcomes related to this assessment were not reported.

People with co-occurring alcohol misuse and mental health problems are often excluded from existing treatment studies [28, 29]. To date, no studies have investigated alcohol misuse, depressive symptomatology, and psychosocial functioning treatment outcomes in a sample of individuals with these co-occurring problems and personality disorder. However, the available evidence suggests that the presence of personality disorders is associated with a greater degree of psychiatric and alcohol use severity [30–32].

Poorer response to treatment for depression has been found among people with co-occurring personality disorders and depression [11]. Higher rates of probable personality disorders (particularly borderline, antisocial, and avoidant personality disorders) have also been linked to lower smoking abstinence rates following group-based CBT, although it is proposed that different personality disorders may impact on initial treatment response and maintenance of abstinence [33]. Treatment outcome studies also report higher rates of attrition [34], lower compliance [32] and poorer outcomes on alcohol use measures at follow-up [15] among individuals with comorbid alcohol misuse and personality disorders. Among clients attending substance misuse services, higher rates of psychopathology (e.g., psychosis, affective and anxiety disorders) and service use have also been reported for those with co-occurring personality disorders [12]. On the other hand, several treatment studies suggest that comorbid personality disorders may not negatively affect alcohol misuse treatment outcomes [35, 36]. The clinical picture may be further complicated by the lack of research on effective evidence-based treatment for personality disorders in this context [37].

Given the relatively high prevalence of co-existing personality disorders, alcohol misuse and depressive symptoms, and the lack of previous research investigating treatment outcomes when these disorders co-occur, it is also important to generate greater understanding of the ways they interact in order to develop effective interventions.

### The current study

We undertook secondary data analysis of composite data from two large randomised controlled trials in an effort to achieve key preliminary insights into the complex interaction between personality disorder cluster scores, alcohol misuse and depressive symptoms. Specifically, we investigated whether baseline personality disorder cluster scores are associated with overall changes in alcohol use, depressive symptoms and functioning, and treatment-related changes at the 6- and the 12-month follow-up, for individuals in treatment for comorbid alcohol misuse and depressive symptomatology.

As hypothesised in the parent studies [28, 38, 39], we expected that participants would display overall improvement between the baseline and follow-up phases on each of the treatment outcome measures. Specifically, we hypothesised that longer (10-session) interventions would result in greater improvements than a brief intervention (comparison condition), that integrated treatment would have greater benefit than single-focused treatment (because interrelationships between comorbid conditions could be better addressed), and that alcohol-focused and depression-focused treatments would have greater impacts on changes in alcohol use and depressive symptoms, respectively. Of particular relevance for the current paper, we also hypothesised that across these outcomes there would be less improvement among participants with higher personality disorder cluster scores, given that specific treatment strategies relating to these issues were not provided in any intervention.

## Methods

### Data sources

This study combined data from two randomized controlled clinical trials co-ordinated by the Centre for Brain and Mental Health Research, University of Newcastle, New South Wales, Australia. Study 1, the Self-Help for Alcohol/other drug use and DEpression (SHADE) project, included 273 participants with comorbid depressive and drug and alcohol problems [39]. Study 2, the Depression and Alcohol Integrated and Single-focused Interventions (DAISI) project, recruited 284 participants with comorbid depressive symptoms and alcohol misuse [28]. Referrals for both studies were accepted from a broad range of sources, including self-referral and referrals by health professionals (e.g., public drug treatment and mental health outpatient clinics, general practices, and non-government support agencies). See Baker et al. [28, 38] and Kay-Lambkin et al. [39] for further details, including full descriptions of the interventions. Several previous reports have also utilised combined SHADE/DAISI datasets, including analyses examining: associations with tobacco smoking [40], hopelessness and suicidal ideation [41, 42]; and the psychometric properties of the Drug Use Motives Questionnaire [43]; see Handley et al. [41]; Section 2.2 for further comment on the rationale for combining these data sources.

Across the two parent studies, a range of manualised MI/CBT based interventions designed to reduce alcohol consumption and/or improve depressive symptoms were delivered. These included a single 90-min brief integrated intervention (BI), followed by either: 1) no further treatment; or 9 further sessions of 2) integrated (alcohol- and depression-focused) therapy (delivered either by a therapist or computer program), 3) alcohol-focused therapy, or 4) depression-focused therapy; in addition, a person-centred (supportive counselling) therapy was offered as a control condition in the SHADE study. Participants provided informed consent and received $20 reimbursement on each assessment occasion (i.e., baseline and follow-up assessments, but not treatment sessions).

### Design and participants

This study involved a secondary analysis of a subset of data from the two randomised controlled clinical trials described above. Subset inclusion criteria for the current study were: (i) hazardous alcohol consumption in the 12 months before baseline (≥ an average of four 10 g ethanol drinks per day for men, ≥ two per day for women); (ii) a BDI-II score ≥ 17; and (iii) assignment to a therapy that specifically targeted alcohol misuse and/or depressive symptoms; consequently SHADE study participants receiving the person-centred therapy intervention were not included in the current study, nor were participants with only cannabis related substance use problems. Among those who were eligible for the current analysis (*N* = 398), the majority had a DSM-IV alcohol dependence disorder (347/379, 92 %) and/or major depression (303/391, 78 %) during the last 12 months.

In addition, potential participants were excluded from the current analysis if they had insufficient data for the primary measures, including: (i) missing personality disorder data at baseline; or (ii) an absence of outcome data at 6- or 12-month follow-up (i.e., none of the primary outcome measures at any follow-up: alcohol consumption, depressive symptomatology or global level of functioning).

### Measures

A description of the full set of assessments employed across the parent studies and the rationale for their use have been reported elsewhere [28, 38, 39]. The current analysis focused on the following subset of measures.

#### Substance misuse, depressive symptoms, and functioning

A range of measures were used to quantify the duration and severity of existing conditions and measure primary outcomes. *The Structured Clinical Interview for DSM-IV-TR* (SCID) [44] was used at baseline to diagnose alcohol dependence and abuse and major depressive episodes in the last 12 months. The *Opiate Treatment Index* (OTI) [45] estimated the average occasions of daily alcohol use in the previous month at baseline and follow-up. Depressive symptoms were assessed at baseline and follow-up using the *Beck Depression Inventory II* (BDI-II) [46]*.* The *Global Assessment of Functioning* scale (GAF) [47] provided a clinician-rated indicator of functioning at baseline and follow-up.

#### International Personality Disorder Examination Questionnaire (IPDEQ) [48]

The parent studies screened participants at baseline for possible Axis II personality disorders using the International Personality Disorder Examination Questionnaire (IPDEQ). The World Health Organization developed the IPDEQ self-report screener based on the International Personality Disorder Examination (IPDE) [48], which is a semi-structured clinician administered interview. The 59-item version of the IPDEQ screener includes items assessing the nine ICD-10 personality disorders: Cluster A – paranoid and schizoid; Cluster B – dissocial, impulsive, borderline and histrionic; and Cluster C – anankastic, anxious and dependent. This instrument was chosen over other possible personality disorder screeners as it is relatively short, yet has been shown to have satisfactory psychometric properties [20]. The current analysis used the IPDEQ cluster scores for each participant, as per the dimensional scoring method described in Lewin et al. [20]. This level of analysis has been shown to have greater predictive power for continuous treatment outcomes than simple categorical assignments [20, 49]. For the current analysis, higher dimensional IPDEQ scores indicate a greater likelihood of personality disorder; other researchers have also used the IPDEQ screener as an indicator of probable personality disorder e.g., [33].

### Data analysis

Data were analysed using SPSS for Windows (version 19; Chicago, IL., USA). Pearson correlations were used to examine simple associations among continuous measures. Single sample t-tests were used to compare IPDEQ profiles with national survey data, while generalized estimating equations were used to examine overall changes between baseline and follow-up phases at 6- and 12-months.

For the major analyses, the primary outcome measures were expressed as change from baseline (i.e., follow-up phase minus baseline scores). Notwithstanding, our focus here was essentially on clinical change at discrete time points, with longer-term benefits (i.e., changes at 12-months) seen as the primary clinical outcome point, and intermediate benefits (i.e., changes at 6-months) viewed as being of lesser importance. Two-step hierarchical linear regressions were used to assess the contributions of socio-demographic, personality disorder and treatment related predictors to change (from baseline) at 6- and 12-months, whilst controlling for baseline scores for the set of outcome measures. Three planned (Helmert) orthogonal treatment condition (TC) contrasts were included at Step 1 in each of these analyses: Contrast TC1, BI vs. 10 sessions (i.e., Group 1 vs. the other 3 groups); Contrast TC2, integrated- vs. single-focused (i.e., Group 2 vs. the last 2 groups); and Contrast TC3, alcohol- vs. depression-focused (i.e., Group 3 vs. the last group); this approach parallels that reported previously for the DAISI trial [38]. All interaction variables were based on continuous standardised scores for the selected predictors (i.e., product variables) and were entered at Step 2 in the regression analyses. To aid interpretation, significant interactions were examined visually by dividing each of the component predictor variables into three approximately equal sub-groups (e.g., Low, Medium and High) and plotting mean scores for the resulting cross-tabulated subgroups for the outcome variables of interest.

Importantly, separate regression models were examined using overall IPDEQ scores and associated interactions; parallel analyses substituted the set of individual cluster scores and associated interactions, providing an opportunity to identify their unique contributions to prediction (i.e., whilst controlling for the other clusters). As a partial control for the number of statistical tests, the threshold for significance was set at *p* < 0.01; however, trends at *p* < 0.05 are also reported.

## Results

### Baseline characteristics

**Fig. 1:**
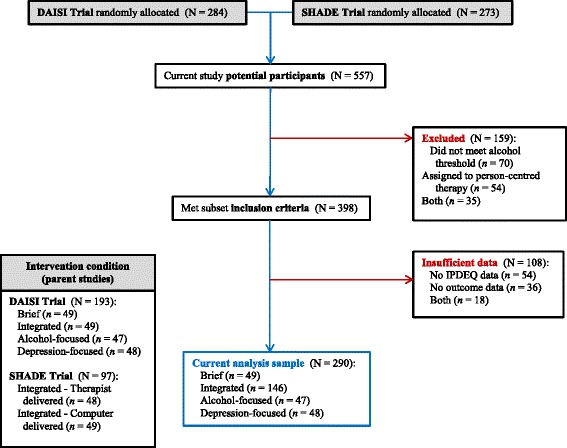
Flow of participants through the parent studies and current analysis

The pattern of participation in the parent studies and current analysis is summarized in Fig. 1. There were 398 eligible participants (i.e., who met the subset inclusion criteria), of whom 290 (73 %) were retained in the current analysis. Among those with insufficient data (*N* = 108), 54 did not have baseline personality disorder data, 36 did not have any outcome data for at least one of the follow-up assessments, and 18 failed to meet both of these criteria. There were no significant differences in baseline characteristics between those who were retained in the current analysis and those with insufficient data; however, the latter subgroup attended fewer treatment sessions (2.98 vs. 5.40, t_(391)_ = 5.30, *p* < .001).

Baseline demographic, symptomatology and substance use data for the selected sample is presented in Table 1. The mean age was 44.58 years and just over half (55 %) were men. Participants had left school at a mean age of 16.18 years and half (54 %) were receiving welfare support at baseline. They averaged 15.08 years of age when they first used alcohol and reported consuming 10.50 standard drinks per day in the month before assessment, well in excess of national recommended guidelines [50]. Participants’ mean BDI-II score was also indicative of severe depression (>30).

**Table 1:** Baseline characteristics of the selected sample (*N* = 290)

Characteristic	Mean (SD, range) or N (%)
Demographic characteristics	
Age (years)	44.58 (10.54, 20-73)
Gender - Male	160/290 (55.2 %)
Country of birth - Australia	236/289 (81.7 %)
Marital status - Single, never married	77/289 (26.6 %)
Children – One or more children	201/289 (70.6 %)
Age left school (years) (*N* = 287)	16.18 (1.32, 12-21)
Post-school qualification	209/280 (74.6 %)
Receiving welfare support	155/288 (53.8 %)
Current symptomatology	
Alcohol Use Disorders (AUDIT) total	26.32 (6.77, 10-40)
Beck Depression Inventory (BDI-II) total (*N* = 285)	30.92 (8.72, 17-55)
Global Assessment of Functioning (GAF) score (*N* = 275)	56.6 (10.32, 25-75)
SCID Axis 1 diagnosis – during last 12 months	
Alcohol abuse only	10/288 (3.5 %)
Alcohol dependence (with abuse)	263/288 (91.3 %)
Major depressive disorder	219/288 (76 %)
Mean occasions of use per day (last month; OTI)	
Alcohol	10.50 (8.14, 0.17-68)
Cannabis	1.35 (5.89, 0-70)
Tobacco	10.81 (12.98, 0-50)
Substance use history – age first used (years)	
Alcohol (*N* = 272)	15.08 (4.26, 2-48)
Cannabis (*N* = 221)	18.94 (7.01, 8-56)
Tobacco (*N* = 247)	15.34 (4.52, 6-43)

*Note*: *SCID* Structured Clinical Interview for DSM-IV-TR, *OTI* opiate treatment index; *DUMQ* Drug Use, Motives questionnaire

#### Personality disorder characteristics

As shown in Table 2, all three mean dimensional scores and the overall personality disorder score for the IPDEQ were significantly higher for participants in the current sample than for those in the national sample reported in Lewin et al. [20]; in each case, the IPDEQ means for the current sample were approximately twice those of the national sample. Correlations between IPDEQ dimensional scores were of a similar magnitude between the three clusters (Clusters A and B: *r* = 0.40; Clusters A and C: *r* = 0.44; and Clusters B and C: *r* = 0.34). These correlations were also comparable to the subset IPDEQ values reported in Lewin et al. [20], based on clinical data from substance users in two community based intervention studies, including 130 people with psychosis and comorbid substance use problems [51] and 155 regular amphetamine users [52].

**Table 2:** Comparisons with IPDEQ dimensional scores from national sample (*N* = 290)

Cluster	IPDEQ dimensional scoring		t	df	Sig. (2-tailed)
	Current analysis	National sample			
	Mean (SD, SE)	Mean (SE)			
Cluster A	0.41 (0.155, 0.009)	0.22 (0.002)	20.57	288	*p* < .001
Cluster B	0.37 (0.155, 0.009)	0.15 (0.002)	24.18	289	*p* < .001
Cluster C	0.49 (0.177, 0.010)	0.22 (0.002)	25.56	289	*p* < .001
Overall PD	0.42 (0.126, 0.007)	0.19 (0.001)	31.09	289	*p* < .001

*Note*: *IPDEQ* International Personality Disorder Examination Questionnaire; *t* single sample *t*-test comparing mean scores for current study against national sample means

### Treatment outcome measures

#### Overall changes in primary outcomes between phases

Estimated mean changes in OTI alcohol scores, BDI-II scores and GAF scores between phases are shown in Table 3. Overall, there was a significant reduction in alcohol use (OTI score) and depressive symptomatology (BDI-II total score) and an improvement in functioning (GAF score) at the 6- and the 12-month follow-ups, relative to baseline. However, improvements between 6- and 12-months were modest, with only changes in depression scores reaching statistical significance (*p* = 0.008). As detailed in Table 3, for all three measures, the observed patterns could also be described as ‘curvilinear’ (i.e., significant linear and quadratic components of change).

**Table 3:** Estimated mean changes between phases for alcohol misuse, depression, and functioning (*N* = 290)

Phase comparison	Alcohol use (per day) (OTI)			Beck Depression Inventory (BDI-II)			Global Assessment of Functioning (GAF)		
	Est. mean change (SD)	W^2^	Sig. (2-tailed)	Est. mean change (SD)	W^2^	Sig.(2-tailed)	Est. mean change (SD)	W^2^	Sig. (2-tailed)
6-months vs. Baseline	-4.94 (9.98)	70.97	*p* < .001	-11.70 (13.87)	205.58	*p* < .001	6.71 (14.71)	59.90	*p* < .001
12-months vs. Baseline	-5.19 (9.89)	79.49	*p* < .001	-13.72 (13.91)	281.32	*p* < .001	8.22 (14.75)	89.61	*p* < .001
12-months vs. 6-months	-0.24 (7.09)	0.34	.563	-2.02 (13.04)	6.98	.008	1.52 (13.88)	3.44	.064

*Note*: Change scores are expressed as the subsequent phase minus the earlier phase. Estimated mean changes and Wald chi-square (W^2^) statistics are from generalized estimating equations utilising all available data. Expressing these changes across phases in orthogonal polynomial terms (as opposed to pair-wise comparisons): all of the linear components of change were statistically significant (with equivalent W^2^ statistics to the 12-months vs. Baseline comparisons), as were all of the quadratic components of change (OTI: W^2^ = 31.65, *p* < .001; BDI-II: W^2^ = 50.94, *p* < .001; GAF: W^2^ = 12.91, *p* < .001)

#### Predictors of change in alcohol use (OTI)

The predictors of OTI change scores at the 6- and the 12-month follow-up are shown in Table 4. As is often the case, baseline OTI scores were strongly associated with OTI change scores at 6- or 12-month follow-up (β = -.790, -.789 respectively); that is, higher baseline scores were associated with more marked improvements. There was also a tendency for those with poorer baseline functioning (GAF) to display greater reductions in alcohol use by 6-months (β = .094), and for females to experience greater reductions in alcohol consumption at 12-months than males (β = -.104). Baseline IPDEQ scores were not predictive of change in alcohol consumption at either follow-up phase. However, among the single-focused treatments, the alcohol-focused intervention tended to produce greater short-term benefits (contrast TC3 at 6-months, β = -.091). There were no significant interaction effects in the alcohol change analyses.

**Table 4:** Predictors of change in alcohol use from baseline: 6- and 12-month outcomes (*N* = 237 and 222)

		Outcome: Change in Alcohol use (OTI) at 6-months				Outcome: Change in Alcohol use (OTI) at 12-months			
	Predictor	Simple correlation	∆R^2^	Standardized regression weight	Sig.	Simple correlation	∆R^2^	Standardized regression weight	Sig.
Step 1			.655				.652		
	Age	-.051		.025	.552	.019		.082	.066
	Gender (M = 1, F = 2)	-.063		-.053	.190	-.160		-.104	.015*
	Baseline alcohol use (OTI)	-.783		-.790	< .001	-.781		-.789	< .001
	Baseline depression (BDI-II)	.072		-.043	.314	.055		-.045	.308
	Baseline functioning (GAF)	-.130		-.094	.027*	-.096		-.084	.060
	*Baseline IPDEQ:*								
	Cluster A	-.013		-.033	.473	.003		.019	.696
	Cluster B	.046		.077	.104	.035		.069	.160
	Cluster C	.055		.056	.205	.041		.025	.603
	Overall score^a^	.045		.077	.073	.039		.086	.055
	*Treatment condition (TC) contrasts:*								
	TC1: Brief vs. 10 sessions	.091		.078	.054	.045		.012	.767
	TC2: Integrated- vs. single-focused	-.041		-.027	.501	-.046		-.056	.182
	TC3: Alcohol- vs. depression-focused	-.109		-.091	.023*	-.094		-.064	.126
Step 2			.007				.015		
	Interactions between cluster scores and TC contrasts (m = 9)								
	Interactions between IPDEQ overall score and TC contrasts (m = 3)^a^								
			(R^2^ = 0.662)				(R^2^ = 0.667)		

*Note*: IPDEQ, International Personality Disorder Examination Questionnaire. Change scores = follow-up phase minus baseline; *Trend (*p* < .05). ^a^From a separate hierarchical regression including IPDEQ overall score (Step 1) and associated interactions (Step 2); only significant or trend level interactions are reported (m = number of interactions examined)

#### Predictors of change in depressive symptomatology (BDI-II)

The predictors of BDI-II change at the 6- and the 12-month follow-up are shown in Table 5. Once again, higher baseline BDI-II scores were associated with more marked reductions in BDI-II scores at 6- and 12-month follow-up (standardised regression weights, β = -.454, -.451 respectively). On the other hand, higher baseline alcohol scores tended to be associated with less improvement in depression at 6-months (β = .117). Higher baseline IPDEQ Cluster C scores were predictive of smaller improvements in BDI-II depressive symptomatology at 6- and 12-month follow-up (β = .194, .187 respectively). This relationship was also reflected in the association between baseline IPDEQ overall scores and BDI-II changes at 6- and 12-month follow-up (β = .215, .216 respectively).

**Table 5:** Predictors of change in depression from baseline: 6- and 12-month outcomes (*N* = 236 and 219)

		Outcome: Change in Depression (BDI-II) at 6-months				Outcome: Change in Depression (BDI-II) at 12-months			
	Predictor	Simple correlation	∆R^2^	Standardized regression weight	Sig.	Simple correlation	∆R^2^	Standardized regression weight	Sig.
Step 1			.245				.302		
	Age	.052		.026	.677	.030		.011	.868
	Gender (M = 1, F = 2)	-.062		.021	.730	-.100		-.009	.888
	Baseline alcohol use (OTI)	.165		.117	.048*	.127		.096	.106
	Baseline depression (BDI-II)	-.397		-.454	< .001	-.415		-.451	< .001
	Baseline functioning (GAF)	.007		-.086	.174	.014		-.068	.292
	*Baseline IPDEQ:*								
	Cluster A	.077		.060	.377	.101		.110	.108
	Cluster B	.011		.018	.798	-.022		-.032	.653
	Cluster C	.144		.194	.003	.148		.187	.005
	Overall score^a^	.102		.215	.001	.095		.216	0.001
	*Treatment condition (TC) contrasts:*								
	TC1: Brief vs. 10 sessions	.127		.090	.135	.248		.216	< .001
	TC2: Integrated- vs. single-focused	-.016		.002	.972	-.061		-.012	.840
	TC3: Alcohol- vs. depression-focused	-.045		-.040	.504	-.096		-.089	.134
Step 2			.036				.059		
	Interactions between cluster scores and TC contrasts (m = 9)								
	Cluster A x TC1					-.100		-.160	.015*
	Cluster A x TC2					-.143		-.166	.019*
	Interactions between IPDEQ overall score and TC contrasts (m = 3)^a^								
	Overall score x TC2					-.107		-.138	.022*
			(R^2^ = 0.280)				(R^2^ = 0.360)		

*Note*: IPDEQ, International Personality Disorder Examination Questionnaire. Change scores = follow-up phase minus baseline; *Trend (*p* < .05). ^a^From a separate hierarchical regression including IPDEQ overall score (Step 1) and associated interactions (Step 2); only significant or trend level interactions are reported (m = number of interactions examined)

**Fig. 2:**
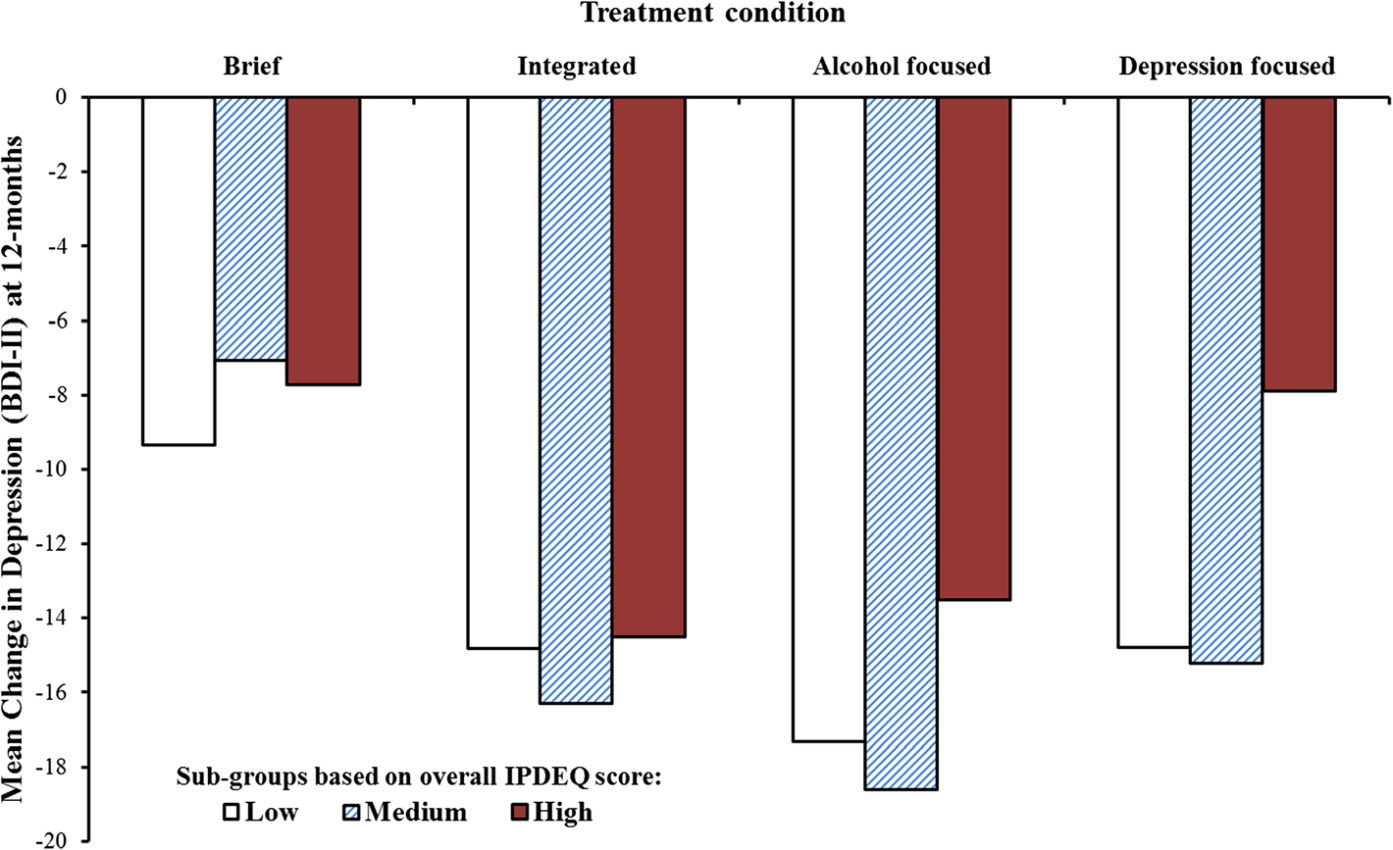
Change in depression (BDI-II) at 12-months by treatment condition and IPDEQ sub-group (*N* = 231)

There were no significant treatment group differences in BDI-II change scores at 6-month follow-up. However, longer treatments produced more marked improvements in depression at 12-months, relative to the BI condition (contrast TC1, β = .216). As shown in Table 5, there were no significant interaction effects at 6-months, although there were three trend level interaction effects at 12-months. The differential benefit at 12-months of the longer interventions was less marked for those with higher Cluster A scores (Cluster A x TC1, β = -.160). Moreover, among the longer interventions, single-focused (vs. integrated) interventions tended to be relatively less effective at 12-months for those with higher Cluster A scores (Cluster A x TC2, β = -.166), and for those with higher IPDEQ overall scores (Overall score x TC2, β = -.138); this latter interaction effect is described more fully in the *Discussion* (and illustrated in Fig. 2).

#### Predictors of change in functioning (GAF)

The prediction of GAF change scores at the 6- and the 12-month follow-up is presented in Table 6. Higher GAF scores at baseline (i.e., better functioning) were associated with smaller improvements in functioning at 6- and 12-month follow-up (β = -.527, -.500 respectively). Additionally, higher baseline IPDEQ overall scores were associated significantly with less improvement in GAF scores at 6- and 12-month follow-up (β = -.231, -.244 respectively); and, at 12-months this effect was due primarily to the influence of baseline IPDEQ Cluster C and Cluster A scores (β = -.108, -.164 respectively). As with change in BDI-II scores, longer treatments produced more marked improvements in functioning at 12-months, relative to the BI condition (contrast TC1, β = -.164). There was also a tendency for greater differential benefit in functioning at 6-months from integrated interventions (vs. single-focused ones) for those with lower Cluster A scores (Cluster A x TC2, β = -.143).

**Table 6:** Predictors of change in functioning from baseline: 6- and 12-month outcomes (*N* = 239 and 221)

		Outcome: Change in Functioning (GAF) at 6-months				Outcome: Change in Functioning (GAF) at 12-months			
	Predictor	Simple correlation	∆R^2^	Standardized regression weight	Sig.	Simple correlation	∆R^2^	Standardized regression weight	Sig.
Step 1			.282				.289		
	Age	-.023		-.009	.877	-.063		-.042	.504
	Gender (M = 1, F = 2)	-.046		-.003	.954	.015		.054	.370
	Baseline alcohol use (OTI)	.008		.002	.971	-.058		-.051	.391
	Baseline depression (BDI-II)	.151		.037	.549	.134		-.026	.672
	Baseline functioning (GAF)	-.480		-.527	< .001	-.435		-.500	< .001
	*Baseline IPDEQ:*								
	Cluster A	-.036		-.088	.198	-.081		-.164	.020*
	Cluster B	-.048		-094	.163	-.022		-.044	.528
	Cluster C	-.092		-.115	.079	-.111		-.108	.005
	Overall score^a^	-.080		-.231	< .001	-.092		-.244	< .001
	*Treatment condition (TC) contrasts:*								
	TC1: Brief vs. 10 sessions	-.052		-.069	.233	-.183		-.164	.007
	TC2: Integrated- vs. single-focused	-.019		.000	.996	.061		.016	.796
	TC3: Alcohol- vs. depression-focused	-.080		-.014	.811	.039		.100	.097
Step 2			.034				.023		
	Interactions between cluster scores and TC contrasts (m = 9)								
	Cluster A x TC2	-.081		-.143	.047*				
	Interactions between IPDEQ overall score and TC contrasts (m = 3)^a^								
			(R^2^ = 0.316)				(R^2^ = 0.312)		

*Note*: IPDEQ, International Personality Disorder Examination Questionnaire. Change scores = follow-up phase minus baseline; *Trend (*p* < .05). ^a^From a separate hierarchical regression including IPDEQ overall score (Step 1) and associated interactions (Step 2); only significant or trend level interactions are reported (m = number of interactions examined)

## Discussion

This study strengthens our understanding of the complexities associated with co-occurring alcohol misuse, depressive symptoms and personality disorder. In broad terms, we sought to investigate whether dimensional personality disorder cluster scores were associated with changes in selected outcomes at the 6- and the 12-month follow-up among outpatients in treatment for comorbid alcohol misuse and depression.

### Treatment effects in this combined dataset

Our first set of hypotheses referred to patterns of change in the outcome measures. As expected, participants’ scores on the OTI, BDI-II and GAF improved across phases, particularly between baseline and the 6-month follow-up. With respect to key treatment related hypotheses derived from the parent studies: longer interventions were more effective than the BI condition at 12-months, contributing to larger reductions in depression and improvements in functioning; collectively, single-focused treatments produced reasonably comparable changes to integrated treatment; and, within the single-focused treatments, alcohol-focused treatment tended to be relatively more effective for alcohol use outcomes, at least in the short-term. These findings are consistent with those reported recently for comparable phases of the DAISI project [38].

The strong associations between baseline scores for the three outcome measures and the corresponding change scores at 6- and 12-months were not surprising, for a range of reasons: because there is a mathematical relationship between difference scores and their constituent parts (e.g., baseline scores are involved in both values); and because samples selected on the basis of more extreme values (here, higher baseline alcohol use and depression scores) would be expected to show some ‘regression to the mean’ effects.

More importantly, as discussed below, this secondary analysis of the combined clinical trials dataset sheds additional light on these treatment effects and associations by examining the contributions of personality disorder cluster scores to the overall prediction of change and to the efficacy of the treatments provided. The presence of some personality disorder profiles negatively impacted on overall change during the follow-up period (primarily Cluster C) as well as treatment-related outcomes (primarily Cluster A), especially in regards to depressive symptoms and psychosocial functioning. These insights have several clinical implications, particularly the potential to inform the development of effective interventions for this comorbid population.

### Limited associations between personality disorder and alcohol outcomes

IPDEQ scores showed limited associations with changes in alcohol use, with no statistically significant effects (see Table 4); that is, there was no evidence that baseline personality disorder severity impacted on subsequent changes in alcohol consumption. Likewise, unlike the change prediction analyses for depression and functioning, there were no significant interactions between IPDEQ scores and treatment condition effects in the alcohol change analyses. This is consistent with earlier research suggesting a limited impact of comorbid personality disorders on alcohol related treatment outcomes [35, 36].

Framing the set of alcohol-related findings more positively: by 6-months, there was an estimated 4.94 drinks per day mean reduction in alcohol consumption; the BI tended to be almost as effective as the longer interventions (with some additional evidence that the alcohol-focused treatment variant was relatively better); and comorbid personality disorder profiles made little contribution. Consequently, from a stepped-care perspective in particular e.g., [53], brief MI/CBT based interventions addressing alcohol misuse should probably be initiated regularly, regardless of personality disorder characteristics (with monitoring of initial treatment responses); similarly, individuals with personality disorders should not be routinely excluded from alcohol misuse treatment.

### Associations between personality disorder, depression and functioning outcomes

Participants with higher baseline IPDEQ overall scores and Cluster C scores experienced smaller improvements in depression at 6- and 12-months (see Table 5). Additionally, the beneficial impacts of longer interventions and single-focused interventions on changes in depression at 12-months tended to be reduced among participants with higher baseline personality disorder cluster scores, particularly Cluster A scores. For example, as illustrated in Fig. 2, the mean benefit associated with the BI was approximately an 8 point reduction in BDI-II scores at 12-months, compared with a more marked, 15 point reduction for the longer interventions; however, participants within the top third of overall IPDEQ scores who were assigned to the depression-focused intervention fared no better than those in the BI condition (i.e., mean improvement around 8 points). Awareness of such possible impacts, by clinicians and their clients, may be an important component of treatment. That is, for individuals with more pronounced personality disorder cluster profiles, simply gaining a better understanding of depressive symptoms and the factors that affect mood, substance misuse and treatment response (as per the integrated intervention condition) may aid recovery.

Consistent with the established impairment in psychosocial functioning for those with personality disorders [31, 54], we also found that higher baseline IPDEQ overall scores were predictive of lower improvement in GAF scores at the 6- and the 12-month follow-up (see Table 6). Furthermore, higher baseline Cluster A and C scores were associated with poorer GAF improvement at 12-month follow-up. Additionally, the impact of single-focused interventions on functioning at 6-months tended to be reduced among participants with higher baseline Cluster A scores; once again, this highlights the potential value of integrated interventions, which may be the preferred approach for individuals with personality disorders.

These observations provide some support for our overall hypothesis that higher personality disorder cluster scores would be associated with poorer outcomes. The social deficits particular to these individuals are not likely to be addressed by standard (non-concurrent) treatments for depressive symptoms and alcohol misuse. Although individuals with personality disorders are more likely to seek treatment for their depression or alcohol use disorder than for their personality pathology [55], it is feasible that individuals with Cluster C profiles may do so for improvements in psychosocial functioning, particularly in regards to interpersonal relationships. This may be less likely for those with socially introverted tendencies, typical of Cluster A personality disorder. Conversely, a recent study suggests that some Cluster A characteristics may reduce the likelihood of smoking relapse among those who have stopped smoking, because of the reduced impact of social pressures [33].

In the current analyses, there was also a tendency for participants with higher baseline alcohol scores to experience less improvement in depression at 6-months, whereas those with poorer baseline functioning experienced greater reductions in alcohol consumption at 6-months. That is, in the short-term, higher alcohol consumption tended to impact in a similar way to the baseline personality disorder scores, in restricting improvements in 6-month depression, while lower baseline functioning scores were associated with greater room for improvement in both alcohol consumption and functioning.

### Limitations

The major limitation of the current study is that it was a secondary data analysis of selected composite data from two randomised controlled trials that did not specifically focus on personality disorder. Therefore, to confirm our findings, a more comprehensive trial is required that includes a clinician administered structured diagnostic assessment for personality disorder. Inclusion of a measure of personality disorder at the conclusion of the follow-up phases would have also been useful. With respect to our current functioning measure, it should also be acknowledged that the GAF has been removed from DSM-5 in favour of more comprehensive assessments of disability and functioning [56]; however, as noted previously [38], the GAF has been found to be more reliable in research settings than in routine clinical practice [57].

Utilisation of separate analyses of change scores at the 6-month and the 12-month follow-ups also raises some potential statistical/methodological concerns, since such outcome scores would be expected to be correlated (and other, more powerful statistical approaches might be possible); however, as noted earlier, for the current analyses change at 12-months was regarded as the primary clinical outcome point. For researchers interested in longitudinal profiles across multiple time points (e.g., linear and non-linear components of change) and their predictors and mediators, different analytical approaches would be preferable, which simultaneously consider data from all phases e.g., [58].

Although we observed significant improvement from baseline in OTI, BDI-II and GAF scores, it should also be acknowledged that 12-month impairment remained substantial; suggesting that the scope and intensity of our interventions needs to be revisited. For example, mean alcohol consumption per day at 12 months (5.32 drinks) was still well above recommended levels, and mean BDI-II scores at 12 months (17.2) were indicative of mild-moderate depression, albeit at the threshold for entry to the parent studies.

It should also be noted that all of the interaction effects detected were at trend significance level (*p* < 0.05); consequently, they await replication elsewhere. On the other hand, studies often lack statistical power to detect complex interactions (in this instance, changes over time by dimensional personality disorder cluster scores by treatment condition effects). However, in the 12-month depression analyses, the (Step 2) interaction effects added 5.9 % to the explained variance (see Table 5, and Fig. 2), suggesting that this effect, in particular, may be worthy of closer investigation.

## Conclusions

In the current analyses (within the combined dataset), longer (10-session) psychological interventions appeared to be more effective in the longer-term (at 12-months), especially for changing depressive symptoms and improving functioning. Moreover, integrated interventions were also relatively more effective than single-focused ones for individuals with higher personality disorder cluster scores. Consequently, longer integrated interventions may be a sensible general strategy for this subgroup, perhaps delivered within a stepped-care framework [53]. However, the observed moderate contributions of higher personality disorder cluster scores to lower improvements in depression and functioning (at 6- and 12-months) also suggests that we need further refinements to our therapeutic approach.

At the very least, adjunctive components need to be incorporated into our integrated therapy programs to more fully engage individuals with particular personality disorder cluster profiles, in an attempt to counteract the potentially negative consequences associated with these conditions, perhaps with a specific focus on emotion or affect regulation and coping strategies. An integrated framework may also provide opportunities to tailor some intervention components to the characteristics and needs of individual participants. We also encourage researchers and clinicians to more actively consider the influence of particular personality disorder clusters in assessing, treating and monitoring individuals engaged in treatment for co-occurring alcohol misuse and depression.

## Abbreviations

BDI-II, Beck Depression Inventory – Version 2; CBT, cognitive behaviour therapy; Cluster A, paranoid & schizoid personality disorders (ICD-10); Cluster B, dissocial, impulsive, borderline & histrionic personality disorders (ICD-10); Cluster C, anankastic, anxious & dependent personality disorders (ICD-10); DAISI, Depression and Alcohol Integrated and Single-focused Interventions (Project); DSM-IV, Diagnostic and Statistical Manual for Mental Disorders – 4^th^ Edition; GAF, The global assessment of functioning scale; ICD-10, International Classification of Diseases – 10^th^ Edition; IPDE, International Personality Disorder Examination; IPDEQ, International Personality Disorder Examination Questionnaire; MI, motivational interviewing; OTI, opiate treatment index; SCID, The Structured Clinical Interview for DSM-IV-TR; SHADE, Self-Help for Alcohol/other drug use and Depression (Project)

## References

[1] NSW Health Department (2000). The management of people with a co-existing mental health and substance use disorder.

[2] Mills KL, Deady M, Proudfoot H, Sannibale C, Teesson M, Mattick R, Burns L (2009). Guidelines on the management of co-occurring alcohol and other drug and mental health conditions in alcohol and other drug treatment settings.

[3] Slade T, Johnston A, Oakley Browne MA, Andrews G, Whiteford H (2009). 2007 national survey of mental health and wellbeing: methods and key findings. Aust NZ J Psychiatry.

[4] Slade T, Johnston A, Teesson M, Whiteford H, Burgess P, Pirkis J, Saw S (2009). The mental health of Australians 2: report on the 2007 national survey of mental health and wellbeing.

[5] Pennay A, Cameron J, Reichert T, Strickland H, Lee NK, Hall K, Lubman DI (2011). A systematic review of interventions for co-occurring substance use disorder and borderline personality disorder. J Subst Abuse Treat.

[6] Jackson HJ, Burgess PM (2000). Personality disorders in the community: A report from the Australian National Survey of Mental Health and Wellbeing. Soc Psychiatry Psychiatr Epidemiol.

[7] Grilo CM, Walker ML, Becker DF, Edell WS, McGlashan TH (1997). Personality disorders in adolescents with major depression, substance use disorders, and coexisting major depression and substance use disorders. J Consult Clin Psychol.

[8] Reich JH, Green AI (1991). Effect of personality disorders on treatment outcome. J Nerv Ment Dis.

[9] Trull TJ, Sher KJ, Minks-Brown C, Durbin J, Burr R (2000). Borderline personality disorder and substance use disorders: a review and integration. Clin Psychol Rev.

[10] Hirschfeld RMA (1999). Personality disorders and depression: comorbidity. Depress Anxiety.

[11] Newton-Howes G, Tyrer P, Johnson T (2006). Personality disorder and the outcome of depression: meta-analysis of published studies. Br J Psychiatry.

[12] Bowden-Jones O, Iqbal MZ, Tyrer P, Seivewright N, Cooper S, Judd A, Weaver T, the Cosmic study team (2004). Prevalence of personality disorder in alcohol and drug services and associated comorbidity. Addiction.

[13] Grant B, Stinson F, Dawson D, Chou S, Dufour M, Compton W, Pickering R, Kaplan K (2004). Prevalence and co-occurrence of substance use disorders and independent mood and anxiety disorders: results from the National Epidemiologic Survey on Alcohol and Related Conditions. Arch Gen Psychiatry.

[14] Grant BF, Hasin DS, Stinson FS, Dawson DA, June RW, Goldstein B, Smith SM, Saha TD, Huand B (2005). Prevalence, correlates, co-morbidity, and comparative disability of DSM-IV generalized anxiety disorder in the USA: Results from the National Epidemiologic Survey on Alcohol and Related Conditions. Psychol Med.

[15] Sher KJ, Trull TJ (2002). Substance use disorder and personality disorder. Curr Psychiatry Rep.

[16] Paris J (2004). Gender differences in personality traits and disorders. Curr Psychiatry Rep.

[17] Gianoli MO, Jane JS, O’Brien E, Ralevski E (2012). Treatment for comorbid borderline personality disorder and alcohol use disorders: a review of the evidence and future recommendations. Exp Clin Psychopharmacol.

[18] Skodol AE (2012). Personality disorders in DSM-5. Annu Rev Clin Psychol.

[19] Furnham A, Milner R, Akhtar R, Fruyt FD (2014). A review of the measures designed to assess DSM-5 personality disorders. Psychology.

[20] Lewin TJ, Slade T, Andrews G, Carr VJ, Hornabrook CW (2005). Assessing personality disorders in a national mental health survey. Soc Psychiatry Psychiatr Epidemiol.

[21] Slade T (2007). The descriptive epidemiology of internalizing and externalizing psychiatric dimensions. Soc Psychiatry Psychiatr Epidemiol.

[22] Andrews G (2004). Management of Mental Disorders.

[23] Brown RA, Evans MD, Miller IW, Burgess ES, Mueller TI (1997). Cognitive-behavioral treatment for depression in alcoholism. J Consult Clin Psychol.

[24] Cornelius JR, Douaihy A, Bukstein OG, Daley DC, Wood SD, Kelly TM, Salloum IM (2011). Evaluation of cognitive behavioral therapy/motivational enhancement therapy (CBT/MET) in a treatment trial of comorbid MDD/AUD adolescents. Addict Behav.

[25] Kay-Lambkin PIDJ, Baker AL, Lewin TJ, Carr VJ (2009). Computer-based psychological treatment for comorbid depression and problematic alcohol and/or cannabis use: A randomized controlled trial of clinical efficacy. Addiction.

[26] Lydecker KP, Tate SR, Cummins KM, McQuaid J, Granholm E, Brown SA (2010). Clinical outcomes of an integrated treatment for depression and substance use disorders. Psychol Addict Behav.

[27] Kelly TM, Daley DC, Douaihy AB (2012). Treatment of substance abusing patients with comorbid psychiatric disorders. Addict Behav.

[28] Baker AL, Kavanagh DJ, Kay-Lambkin FJ, Hunt SA, Lewin TJ, Carr VJ, Connolly J (2010). Randomized controlled trial of cognitive-behavioural therapy for coexisting depression and alcohol problems: Short-term outcome. Addiction.

[29] Ekleberry SC (2008). Integrated Treatment for Co-Occurring Disorders: Personality Disorders and Addiction.

[30] Bakken K, Landheim A, Vaglum P (2007). Axis I and II disorders as long-term predictors of mental distress: a six-year prospective follow-up of substance-dependent patients. BMC Psychiatry.

[31] Langas A-M, Malt U, Opjordsmoen S (2012). In-depth study of personality disorders in first-admission patients with substance use disorders. BMC Psychiatry.

[32] Ross S, Dermatis H, Levounis P, Galanter M (2003). A comparison between dually diagnosed inpatients with and without Axis II comorbidity and the relationship to treatment outcome. Am J Drug Alcohol Abuse.

[33] Piñeiro B, Fernández del Río E, López-Durán A, Martínez Ú, Becoña E (2013). The association between probable personality disorders and smoking cessation and maintenance. Addict Behav.

[34] Reich JH, Vasile RG (1993). Effect of personality disorder on the treatment outcome of Axis I conditions: An update. J Nerv Ment Dis.

[35] Longabaugh R, Rubin A, Malloy P, Beattie M, Clifford PR, Noel N (1994). Drinking outcomes of alcohol abusers diagnosed as Antisocial Personality Disorder. Alcohol Clin and Exp Res.

[36] Messina NP, Wish ED, Hoffman JA, Nemes S (2002). Antisocial personality disorder and TC treatment outcomes. Am J Drug Alcohol Abuse.

[37] Verheul R, van den Bosch LMC, Ball SA, Oldham JM, Skodol AE, Bende DS (2005). Substance abuse. Textbook of personality disorders.

[38] Baker AL, Kavanagh DJ, Kay-Lambkin FJ, Hunt SA, Lewin TJ, Carr VJ, McElduff P (2014). Randomized controlled trial of MICBT for co-existing alcohol misuse and depression: Outcomes to 36-months. J Subst Abuse Treat.

[39] Kay-Lambkin FJ, Baker AL, Kelly B, Lewin TJ (2011). Clinician-assisted computerised versus therapist-delivered treatment for depressive and addictive disorders: A randomised controlled trial. Med J Aust.

[40] Kay-Lambkin F, Edwards S, Baker A, Kavanagh D, Kelly B, Bowman J, Lewin T (2013). The impact of tobacco smoking on treatment for comorbid depression and alcohol misuse. Intl J Ment Health Addict.

[41] Handley TE, Kay-Lambkin FJ, Baker AL, Lewin TJ, Kelly BJ, Inder KJ, Attia JR, Kavanagh DJ (2013). Incidental treatment effects of CBT on suicidal ideation and hopelessness. J Affect Disord.

[42] Handley TE, Kay-Lambkin FJ, Baker AL, Lewin TJ, Kelly BJ, Inder KJ, Attia JR, Kavanagh DJ. Investigation of a suicide ideation risk profile in people with co-occurring depression and substance use disorder. J Nerv Ment Dis. 2016. In Press. 10.1097/NMD.0000000000000473 26807880

[43] Thornton LK, Baker AL, Johnson MP, Kay-Lambkin F, Lewin TJ (2012). Reasons for substance use among people with psychotic disorders: Method triangulation approach. Psychol Addict Behav.

[44] First MB, Spitzer RL, Gibbon M, Williams JB (2001). Structured Clinical Interview for DSM-IV-TR axis I disorders (SCID), research version, patient edition.

[45] Darke S, Hall W, Wodak A, Heather N, Ward J (1992). Development and validation of a multi-dimensional instrument for assessing outcome of treatment among opiate users: The Opiate Treatment Index (OTI). Br J Addict.

[46] Beck AT, Steer RA, Brown GK (1996). The Beck Depression Inventory (BDI-II), Second Edition: Manual.

[47] American Psychiatric Association (2000). Global Assessment of Functioning (GAF) Scale.

[48] Loranger AW, Janca A, Sartorius N (1997). The ICD-10 International Personality Disorder Examination (IPDE).

[49] Shea MT, Widiger TA, Klein MH (1992). Comorbidity of personality disorders and depression: Implications for treatment. J Consult Clin Psychol.

[50] National Health and Medical Research Council [NHMRC] (2009). Australian guidelines to reduce health risks from drinking alcohol.

[51] Baker AL, Bucci S, Lewin TJ, Kay-Lambkin F, Constable PM, Carr VJ (2006). Cognitive behavioural therapy for substance use disorders in people with psychotic disorders: Randomised controlled trial. Br J Psychiatry.

[52] Baker AL, Lee NK, Claire M, Lewin TJ, Grant T, Pohlman S, Saunders JB, Kay-Lambkin F, Constable P, Jenner L (2004). Drug use patterns and mental health of regular amphetamine users during a reported ‘heroin drought’. Addiction.

[53] Morley KC, Baillie A, Leung S, Sannibale C, Teesson M, Haber PS. Is specialized integrated treatment for comorbid anxiety, depression and alcohol dependence better than treatment as usual in a public hospital setting? Alcohol Alcohol. 2016;51:402–409. 10.1093/alcalc/agv131 26672793

[54] Skodol AE, Gunderson JG, McGlashan TH, Dyck IR, Stout RL, Bender DS, Grilo CM, Shea MT, Zanarini MC, Morey LC (2002). Functional impairment in patients with schizotypal, borderline, avoidant, and obsessive-compulsive personality disorder. Am J Psychiatry.

[55] Bender DS, Dolan RT, Skodol AE, Sanislow CA, Dyck IR, McGlashan TH, Shea MT, Zanarini MC, Oldham JM, Gunderson JG (2001). Treatment utilization by patients with personality disorders. Am J Psychiatry.

[56] Gold LH (2014). DSM-5 and the assessment of functioning: The World Health Organization Disability Assessment Schedule 2.0 (WHODAS 2.0). J Am Acad Psychiatry Law.

[57] Startup M, Jackson MC, Bendix S (2002). The concurrent validity of the Global Assessment of Functioning (GAF). Br J Clin Psychol.

[58] Gibbons RD, Hedeker D, DuToit S (2010). Advances in analysis of longitudinal data. Annu Rev Clin Psychol.

